# Multivariate Statistical Assessment of Predictors of Firefighters’ Muscular and Aerobic Work Capacity

**DOI:** 10.1371/journal.pone.0118945

**Published:** 2015-03-16

**Authors:** Ann-Sofie Lindberg, Juha Oksa, Henrik Antti, Christer Malm

**Affiliations:** 1 Sports Medicine Unit, Umeå University, Umeå, Sweden; 2 Winternet, Boden, Sweden; 3 Physical Work Capacity-team, Finnish Institute of Occupational Health, Oulu, Finland; 4 Department of Chemistry, Umeå University, Umeå, Sweden; University of Rome, ITALY

## Abstract

Physical capacity has previously been deemed important for firefighters physical work capacity, and aerobic fitness, muscular strength, and muscular endurance are the most frequently investigated parameters of importance. Traditionally, bivariate and multivariate linear regression statistics have been used to study relationships between physical capacities and work capacities among firefighters. An alternative way to handle datasets consisting of numerous correlated variables is to use multivariate projection analyses, such as Orthogonal Projection to Latent Structures. The first aim of the present study was to evaluate the prediction and predictive power of field and laboratory tests, respectively, on firefighters’ physical work capacity on selected work tasks. Also, to study if valid predictions could be achieved without anthropometric data. The second aim was to externally validate selected models. The third aim was to validate selected models on firefighters’ and on civilians’. A total of 38 (26 men and 12 women) + 90 (38 men and 52 women) subjects were included in the models and the external validation, respectively. The best prediction (R^2^) and predictive power (Q^2^) of *Stairs*, *Pulling*, *Demolition*, *Terrain*, and *Rescue* work capacities included field tests (R^2^ = 0.73 to 0.84, Q^2^ = 0.68 to 0.82). The best external validation was for *Stairs* work capacity (R^2^ = 0.80) and worst for *Demolition* work capacity (R^2^ = 0.40). In conclusion, field and laboratory tests could equally well predict physical work capacities for firefighting work tasks, and models excluding anthropometric data were valid. The predictive power was satisfactory for all included work tasks except *Demolition*.

## Introduction

Firefighters have varied duties, including operative tasks (such as fighting fires and rescuing people and animals), service of materials, equipment and vehicles, engage in fire protective work, and within some fire and rescue services: give first aid in case of medical emergency calls.

Work performance is a broad term, has different meanings within different disciplines, and includes several dimensions [1,2]. Some of the dimensions affecting firefighters’ work performance are the use of fire protective clothing [3] and Breathing Apparatus (BA) [4–7], ergonomics and heavy equipment [8–10], increased body temperature [11], the time passing between turn-outs [12], physical work capacity [13–18], emotional stress [19], cognitive abilities [20], work-organizational and psychosocial factors, and perceived work-related strain among firefighters [21,22]. Consequently, physical work capacity is only one of several dimensions affecting firefighters’ work performance. Several work tasks, e.g. carrying equipment up stairs and victim rescue are rated as physically demanding by firefighters [13,22–25], and these work tasks may therefore set high demands on physical work capacity. The load of firefighters’ protective gear reduces relative muscle strength, anaerobic capacity, and maximal aerobic power (VO_2max_: mL·kg^-1^∙min^-1^) [26], theoretically reinforces the importance of a high physical capacity. Consequently, a high aerobic capacity (VO_2max_: L·min^-1^) [7,17,27,28], VO_2max_ (mL·kg^-1^∙min^-1^) [13,17,29,30], as well as muscle strength and endurance [16,18,28,31–36] are of fundamental importance for firefighters’ physical work capacity. A low physical work capacity may reduce the safety of the individual firefighter, the colleague or the victim.

In accordance with the government regulation for permission to execute smoke diving, Swedish firefighters’ must meet physical requirements in a pass or fail test [37]. Upon recruitment, additional physical tests are performed for evaluation of physical work capacity. These physical tests are composed by each individual municipality, may differ between locations and have unknown scientific validity. In Sweden, subjects applying to work as a firefighter do not always have firefighting experience. Consequently, the physical tests have to be valid both for firefighters’ and for civilians’. Significant correlations between firefighters’ physical work capacity and VO_2max_, muscle strength/endurance in the upper [28,31,32,35,36] and lower [31,32,34–36] body have previously been found. However, there is a lack of studies combining simulated firefighting work tasks with laboratory and field-tests for investigation of muscle strength and endurance of firefighters [14,18]. Using simple field tests have been advocated for evaluation of physical work capacity [16,33], but using a combination of field and laboratory tests may increase the validity of executed tests.

When pre-employment tests are used for selection of firefighters, it is important that these tests are valid to physical work capacity. In order to rank and select the physical tests that best predict firefighters’ work capacity, it is necessary to initially include a large number of tests. Anthropometric data, such as percent body fat [34,38] and body mass [34] have previously been found both to affect, and not to affect firefighters’ physical work capacity. Because body composition may or may not affect physical capacity, using anthropometric data as selection criterions may be discriminative, and negatively select a person based on incorrect assumptions.

Statistical methods previously used for development of relevant physical tests for firefighters´ are essentially bivariate and multivariate linear regression. An alternative to these traditional statistical methods is multivariate (MVA) statistical or projection analysis, mainly used within chemo metrics. Multivariate statistical analysis is an appropriate statistical approach to increase the statistical power and facilitate interpretation when the dataset consists of few observations (subjects) and many correlated variables, and a viable complement to bivariate statistics [39]. In addition, data can be both cross-validated (predictive power on the same subject group) and externally validated (on another subject group), giving additional information of the selected models robustness.

In order to assess firefighters' physical work capacity, the combined effect of physical capacities needs to be taken in to account. To increase accessibility and reduce costs, field-tests rather than laboratory tests are preferred. The first aim of the present study was to evaluate the prediction and predictive power of field and laboratory tests, respectively, on firefighters’ physical work capacity on selected work tasks. Also to study if valid models could be achieved when anthropometric data were excluded. The second aim was to externally validate selected models. The third aim was to validate selected models on firefighters and civilians.

## Method

### Study design

The present study includes two datasets: one training-set and one prediction-set. The training-set was used to select a battery of physical tests for evaluation of firefighters’ physical work capacity. Variables included were obtained from ten laboratory tests, fourteen field tests, three anthropometric variables, and five simulated firefighting work tasks, with a total of 99 variables (Table 1). These physical tests/simulated work tasks were executed over ten non-consecutive, randomized days, each day separated by at least one non-testing day. The inclusion of physical tests was based on [17,18] an attempt to imitate physical demands within firefighting work tasks, with respect to movement, equipment weights, and work duration. Work tasks were included based on a previous study of firefighters’ rated physical demands [22], discussions with an expert group within the Swedish Civil Contingencies Agency (SCCA), as well as previous studies of firefighters’ physical work capacity of our research group [17,18].

**Table 1:** Physical test variables, overview.

Variable-group	Variables included
Anthropometrics	**Body height: m, Body weight: kg, Body Mass Index: kg∙m^−2^**
Laboratory aerobic	*Submaximal treadmill running*: Variables at OBLA and LT: Treadmill speed, % HR_max_, % VO_2max_, VO_2_ mL∙min^−1^ *VO2*_*max*_: mL∙min^−1^ and mL∙kg^−1^∙min^−1^, RER_max_, VE_max_, [La^−^]_b max_
Field aerobic	Cycling at 200 W: % HR_max_, Step-up test: % HR_max_ and Treadmill walking: % HR_max_, Crawling 30 m: s, **Running 3000 m**: **s and s**∙**kg** ^**−1**^, **Rowing 500 m: s**
Laboratory muscle strength/endurance	Maximal and endurance Shoulder flexion and extension and Knee extension and flexion: Nm, Nm %, W, W∙kg^−1^, J. Endurance Shoulder press and Deadlift: N, N %, W, W∙kg^−1^, J. Endurance Trunk extension and flexion: Nm, Nm %, W, W∙kg^−1^, J.
Field muscle strength/ endurance	**Maximal handgrip strength: kg**. Sit ups, Squat, **Bench press**, **Upright barbell row** and Barbell shoulder press: n. Handgrip endurance: s
Laboratory Balance	Postural stability: overall, medial lateral, and anterior posterior stability index
Field muscle power	**Standing broad jump: m**
Work tasks	**Stairs, Pulling, Demolition, Rescue, and Terrain: s.**

The training-set performed all physical tests, the prediction-set performed physical tests in bold style. Some physical tests included several variables. LT: Lactate threshold, OBLA: Onset of blood lactate accumulation, [La-]_b max_: maximal lactate accumulation in blood, s: seconds, s∙kg^−1^: seconds scaled to kg body mass, N: Newton, N %: N scaled to body weight, Nm: Newton meter, Nm %: Nm scaled to body mass, W: Watt, W∙kg^−1^: W scaled to body mass. J: Joule. Work tasks included were Carrying hose baskets up stairs (Stairs), Hose pulling (Pulling), Demolition at or after a fire (Demolition), Victim rescue (Rescue), and Carrying hose baskets over terrain (Terrain).

The prediction-set was used for external validation, and included six field tests, three anthropometric variables, and five simulated firefighting work tasks, with a total of 15 variables (Table 1). Tests were executed over three consecutive days and extracted from our previously published studies [17,18] and our government report (Swedish) that used linear multivariate regression analyses [40].

### Subjects

#### Training-set

After receiving written and verbal explanation of the procedure, 42 subjects, including male full-time firefighters, male part-time firefighters, and civilian men and women with no experience of working as firefighters, volunteered to participate. No female firefighters were available for inclusion. All subjects included in the training-set were also included in previous studies related to the current study, evaluating the aerobic work capacity [17] and muscle strength and endurance, and dynamic balance in firefighters [18]. Due to the extensive data, publications are separated according to aims.

#### Prediction-set

After receiving written and verbal explanation of the procedure, 90 subjects, including male and female full-time firefighters, male and female part-time firefighters, and civilian men and women with no experience of working as firefighters, volunteered to participate. Subjects were recruited from the Fire and Rescue Services in Sweden and by notices at local gyms.

#### Ethics statement

All participants signed an informed consent, stating their ability to execute all parts of the study, and absence of any known diseases affecting physical performance. The Research Ethics Committee for Northern Sweden at Umeå University approved the study on September 22, 2009 (Dnr 09–046M) and the study was conducted in accordance with the WMA Declaration of Helsinki—Ethical Principles for Medical Research Involving Human Subjects 2008.

### Descriptive data and physical tests

All subjects’ filled in a health questionnaire stating absence of any known diseases. Subjects were always dressed in shorts/pants, t-shirt, and training shoes during the physical tests. During the simulated firefighting work tasks, additional clothing was used.

All physical tests and simulated work tasks were performed at maximal capacity (e.g. maximum speed, number of repetitions or force), unless otherwise stated. Every test day including laboratory and field tests started with an appropriate and standardized 5 to 15 min warm up procedure. Simulated work task tests were not preceded with warm up because in a real time situation there is no warm up session prior work. Levels et al. [41] found no significant differences in firefighting work tasks speed in a pre-warming vs. thermo neutral group, during initial work (≈ 10 min).

Subjects were instructed in the procedure of all physical tests and simulated firefighting work tasks, but no familiarizations were scheduled. An extensive description of the method and equipment used in all tests has previously been presented [17,18] and below a summary of the variables used within each test is provided.

A five to ten minutes rest separated physical tests performed by the training-set, all subjects had equal resting period. For the prediction-set, all physical tests were separated by five to 240 minutes rest: field tests were performed in one day with half of the tests in the morning and half of the tests in the afternoon.

#### Descriptive data

Descriptive data included systolic and diastolic arterial blood pressure (mmHg) (TriCUFF:AJ Medical, Stockholm, Sweden), body mass (kg), standing height (m) (SECA GmbH & Co. KG, Hamburg, Germany) and Body mass index (BMI: kg∙m^-2^).

#### Laboratory tests of muscular strength, muscular endurance and balance

The training-set performed concentric isokinetic tests of maximal muscle force on a Biodex Multi-Joint system 3 dynamometer (Biodex medical system, New-York, USA) [18] (Table 1). Total work (J) is the amount of work accomplished for the entire set, average power (W) is a measure of an individual´s ability to produce torque or force within the tests range of motion (ROM) divided by time. Peak torque (Nm) and force (N) were also registered, and also scaled to body mass expressed as either % (N and Nm) or W∙kg^-1^ [42,43]. From bilateral tests, the highest individual performance is presented. *Endurance shoulder press* was performed with one set of 15 rep max at an angular speed of 240°∙sec^-1^ [18]. *Endurance deadlift (floor to knee)* was performed with one set of 15 rep max at an angular speed of 240°∙sec^-1^ [18]. *Maximal and endurance shoulder flexion and extension* were performed with one set of maximal muscle force (5 rep max at an angular speed of 60°∙sec^-1^)[18], followed by one set of muscle endurance (15 rep max at 180°∙sec^-1^) [18]. *Maximal and endurance knee extension and flexion* was performed with one set of maximal muscle force (5 rep max at an angular speed of 60°∙sec^-1^), followed by one set of muscle endurance (30 rep max at 180°∙sec^-1^) [18]. *Endurance trunk flexion and extension* was performed with one set of 15 rep max trunk flexion and extension at 60°∙sec^-1^ [18].

The training-set executed a laboratory test of *Dynamic Stability* on the Biodex Balance system SD (Biodex medical system, New-York, USA) [18] (Table 1).

#### Field tests of muscular strength and endurance

Benches, barbells, a smith machine (Precor, CL Fitness, Sweden), dumbbells, and free weights (Casall Sport AB. Sweden) were used. The weight of barbells, dumbbells, and free weights were controlled with the previously presented SECA scale. A metronome (Korg MA-30 metronome: Korg and Moore, Marburg, Germany) was used for tests performed at a pre-defined speed. Tests were stopped if the required pace or range of motion could not be followed, despite three verbal encouragements for correction. Only correctly performed exercises were counted. All included field tests are presented in Table 1. Tests of muscle endurance were executed with equal barbell weights irrespectively of subject’s sex, age or body weight, because in a real time situation the weight of the protective gear and equipment are equal for all firefighters.

Muscle strength: Both the training-set and prediction-set performed a test of *Maximal handgrip strength* (Grip-D: Eleiko sport AB, Halmstad). The highest performance from three trials on each hand was registered [18].

Muscle endurance: The training-set performed a *Sit-ups* test with a standardized lifting height and speed [18,44], *Endurance handgrip* test, holding a 27.0 kg dumbbell in each hand, a *Squat* test using a Smith machine (22.0 kg barbell) and standardized speed, and an *Barbell shoulder press* test with a 7.5 kg EZ-barbell performed at a standardized speed [18]. Both the training-set and prediction-set performed a *Bench press* test at a standardized speed, using a 30 kg barbell and an *Upright barbell row* test with a 7.5 kg EZ-barbell performed at a standardized speed [18].

Muscle power: Both the training-set and predictions-set performed a *Standing broad jump* test, the best jump out of three performed was registered [18].

#### Laboratory aerobic fitness tests

Laboratory aerobic tests [17] were performed on a treadmill. Continuous measurements of heart rate (HR) (Polar heart rate monitor S810; Polar Electro Oy, Kempele, Finland), oxygen consumption (VO_2_), ventilation (V_E_) and respiratory exchange ratio (RER) were made (Jaeger Oxycon Pro; Care Fusion, San Diego, USA) with Hans Rudolph accessories (Hans Rudolph Inc., Shawnee, Kansas, USA). Fingertip blood lactate was analyzed with Biosen 5130 (EKF-diagnostic, GmbH, Barleben, Germany). The training-set performed a *Submaximal treadmill running* test in order to determine lactate threshold (LT) [45,46] and Onset of blood lactate accumulation (OBLA) [46,47] and a *Maximal treadmill running* test to measure VO_2max_ [17] (Table 1).

#### Field aerobic fitness tests

Field aerobic tests were performed indoors. The training-set performed a submaximal 6 min *Cycling* (Ergomedic, 839 E; Monark Exercise AB, Vansbro, Sweden) test at 200 W using a standardize cadence, and a maximal 30 m *Crawling* test [17]. In addition, dressed in personal protective gear including BA (the total weight of clothing and equipment was 24 ± 0.5 kg) they performed both a submaximal 6 min *Step-test* at a standardized speed, and a submaximal 6 min *Treadmill walking* test at 4.5 km∙h^-1^ and 8° incline [17,37]. Both the training-set and prediction-set performed a maximal *3000 m Running* and a *500 m Rowing* (Concept II: Concept2. Inc., Morrisville, USA) test [17] (Table 1).

#### Simulated work tasks

Both the training-set and prediction-set performed a work task course including *Carrying hose baskets upstairs* (*Stairs*), *Hose pulling (Pulling*), *Demolition at or after a fire* (*Demolition*), and *Victim rescue* (*Rescue*) [17,18]. These tasks were performed in sequence with two minutes of active rest (aimed for moving between the stations) between each work task. Subjects were dressed in a fire emergency jacket, gloves, and BA (19.0 ± 0.5 kg). The *Pulling* work task was slightly different for the training-set and the prediction-set. The training-set used a 70 mm diameter rope, and the prediction-set used a 70 mm diameter water-filled hose. The pulling resistance was equal, as verified by slowly pulling the rope/ hose. In addition, both subject groups performed a *Carrying hose baskets over terrain* (*Terrain*) work task with a total distance of 1600 m [17,18] (Table 1).

### Statistics

Descriptive comparisons between the training-set and the prediction-set were carried out with Statistical Package for the Social Sciences (SPSS) version 20.0 (IBM Corp, Armok, NY, USA), using One Way ANOVA. Physical test capacity comparisons between the training-set and the prediction-set were executed with independent t-test for parametric data, and with the Kruskal-Wallis test for non-parametric data. Data was considered as normally distributed if at least two of three parameters were achieved: skewness and kurtosis ranged within ± 2.58 of standard error, the Shapiro-Wilk´s test was > 0.05 and the Q-Q Plot were normally distributed, visually inspected [48]. Bivariate correlations of work task performances were analyzed with Pearson product-moment correlation coefficient (r).

Multivariate statistical analyses were executed in SIMCA version 13.0 (MKS Umetrics AB, Umeå, Sweden). SIMCA provides options pattern recognition at several levels. All data was mean centered and scaled to unit variance (UV) prior to analysis. Variables that were considered as skewed were log_10_ transformed to normality. To simplify interpretation of the models, variables with a low performance time equaling high performance were converted: 1/variable. These variables were: *Running 3000 m* (s and s∙kg^-1^), *Cycling*, *Treadmill walking*, and *Step-up* (% HR_max_), *Rowing 500 m*, *Crawling*, *Stairs*, *Pulling*, *Rescue*, and *Terrain* (s), and *Dynamic balanc*e.

#### First modeling

The initial multivariate modeling was performed with data from the training-set. The total number of variables obtained from the laboratory and field tests, and anthropometrics were 94 (X = 94) and the total numbers of variables obtained from simulated work tasks were five (Y = 5) (Table 1). For an overview of data, an unsupervised Principal Component Analysis (PCA) was performed and for prediction of the overall fit of the model (R^2^ = 1, the model explains 100% of the variation in the data) and the predictive power (also called cross-validation) of the model (Q^2^) an Orthogonal Projection to Latent Structures (OPLS) was done. The predictive power is a measure of the ability of the model to predict same variation based on cross-validation. We considered models having both an R^2^ and a Q^2^ > 0.60 to be valid.

PCA and OPLS generate a score and loading plot. The score plot represents the projection subjects (observations) and the loading plot the projection of the variables (loadings). The score plot shows correlations between observations, identifies if they are related to each other and if there are any groups or trends. The loading plot shows correlations between variables. Variables with the largest absolute values of projection (p) 1 or/and p2 in the loading plot dominate the projection and are the most influential variable for the distribution of the observations in the score plot. Variables close to each other are positively correlated; variables opposite to each other are negatively correlated. The score and the loading plots are linked, and together they generate an overview of the variation in the data, provides information and explanations regarding sample distributions, e.g. group separations and display variables correlations. In OPLS, X represents the regressor variables (laboratory and field tests, and anthropometric data) and Y represents the response variables (work tasks). For each work task, an OPLS models and separates the systematic variation in X correlated to Y (predictive variation) and the systematic variation uncorrelated to Y (orthogonal variation). To make this report easy to read, mathematical and technical information have been omitted, and for details the reader is referred to other publications [49–51]. For each work task, one model included laboratory tests and anthropometric data (X = 79), and one included field tests and anthropometrics (X = 18). For each model, a stepwise variable selection was executed: First, variables having the lowest Variable Importance for the Projection (VIP) were excluded until an optimal model was obtained. The optimal model included as few variables as possible without a decrease in Q^2^ > 0.05. VIP summarizes the importance of the variables both to explain X and to correlate to Y. VIP- values larger than 1 indicates that the variable is important for the projection, and values lower than 0.5 indicates that the variable is unimportant for the projection. Second, when physical test variables were highly correlated (r ≥ 0.8) to each other, only the variable having the highest VIP to the specific work task was included.

#### External validation

External validation is a rigorous way of testing the predictive performance on an independent set of observations that has not been used in the model building. The external validation of selected physical tests was executed with the prediction-set. In order to achieve the most proper external validation, all X variables represented in the training-set should also be represented in the prediction-set [39].

#### Extended analysis

The selected field-test model was tested with all subjects included (training-set + prediction-set), on firefighters (n = 83) and on civilians (n = 45).

#### Second modeling

With both the training-set and the prediction-set included (n = 128), a new model was created, starting with inclusion of tests performed by both the training-set and the prediction-set (Table 1). The model selected with all subjects included was tested separately on firefighters and on civilians.

## Results

### Subjects

Out of the 42 subjects included in the training-set, 38 subjects completed the study and four subjects dropped out due to their lack of time. All 90 subjects included in the prediction-set completed the study. Subjects included were male full-time firefighters (training-set: n = 10, prediction-set: n = 12), male part-time firefighters (training-set: n = 8, prediction-set: n = 13), civilian men (training-set: n = 8, prediction-set: n = 13), female full-time firefighters (training-set: n = 0, prediction-set: n = 17), female part-time firefighters (training-set: n = 0, prediction-set: n = 23), and civilian women (training-set: n = 12, prediction-set: n = 12). Significant differences between the training-set and the prediction-set were found in physical tests (Table 2).

**Table 2:** Between group differences in descriptive data and physical tests.

Variable	SM	Training-set n = 38	Prediction-set n = 90
**Age (year)**	P	34±9.8	35±9.8
**Height (m)**	P	1.77±0.08	1.74±0.09
**Weight (kg)**	P	78±11.1	76 ±11.2
**BMI (m** ^**3**^)	P	25±2.6	25±2.7
**BP Systolic (mmHg)**	P	130±12.8	126±12.7
**BP diastolic (mmHg)**	P	71±9.9	68±10.6
**3000 m running (s)**	NP	810± 167 ^A^	867±167 ^C^
**3000 m running (s**∙**kg** ^-**1**^)	NP	10.7± 2.3 ^A^	11.6± 3.2 ^C^
**Grip strength (kg)**	NP	57±24.6	43±16.9 *
**Bench press (n)**	NP	47±56	28±23
**Rowing 500 m (s)**	NP	97±18	103±16
**Barbell shoulder lift (n)**	NP	102±79	152±111 *
**Broad jump (cm)**	NP	237±54 ^A^	210±57 *
**Terrain (s)**	NP	690±183	779±199 ^B^
**Stairs (s)**	NP	77±40 ^B^	99±43 *
**Pulling (s)**	NP	17±10 ^A^	35±36 *
**Demolition (s)**	NP	86±50 ^A^	60±41
**Rescue (s)**	NP	22±10 ^A^	27±12 *

Between group differences for subjects included in the training-set (n = 38) and in the prediction-set (n = 90).

^A^ One subjects did not do the test.

^B^ Two subjects did not do/ complete the test.

^C^ Four subjects did not do/complete the test BMI: Body Mass Index. BP: Blood pressure. Investigated work tasks were Carrying hose baskets over terrain (Terrain), Carrying hose baskets up stairs (Stairs), Hose pulling (Pulling), Demolition at or after a fire (Demolition) and Victim rescue (Rescue). Statistical methods (SM): for parametric data (P) mean ± Standard deviation is presented. For non-parametric data (NP) median ± Interquartile range is presented. * p < 0.01.

For variables included both in the training-set and the prediction-set, missing data is presented in Table 2. Additional missing data found in the training-set were for OBLA and LT treadmill speed, and OBLA and LT % HR_max_: n = 4, OBLA % VO_2max_: n = 6 and % HR_max_ at 200 W cycling: n = 5.

### Modeling and external validation

**Fig 1:**
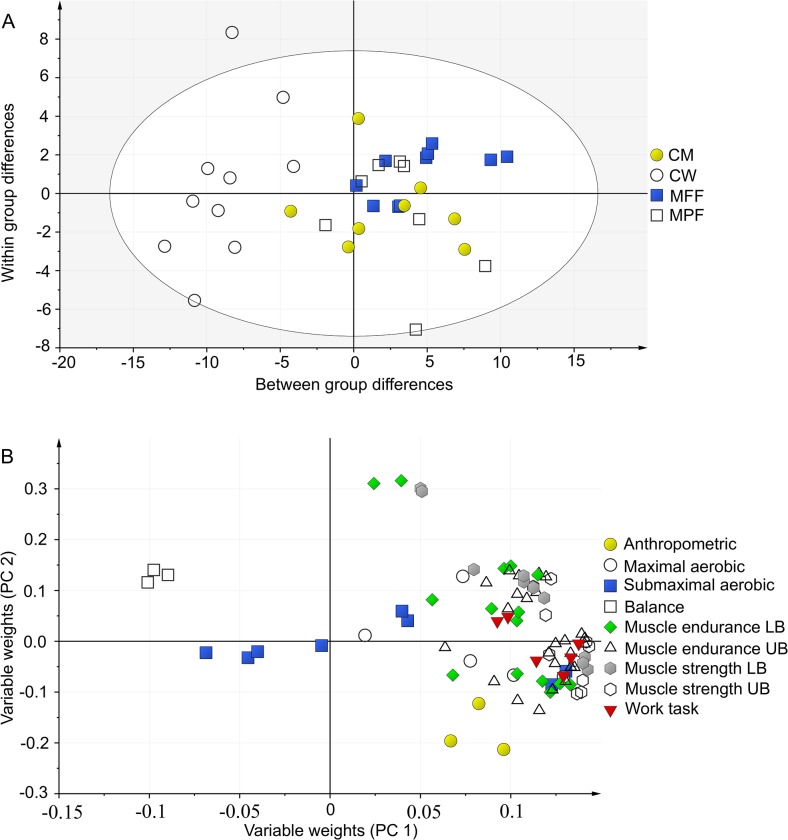
Principal component analysis, laboratory tests and anthropometrics. Score scatter plot (A) of the training-set visualizes the main variation in the data, here seen as between-group differences, and within-group differences. CM: Civilian Men, CW: Civilian Women, MFF: Male Full time Firefighters, MPF: Male Part time Firefighters, n = 36. Loading scatter plot (B: X = 79, Y = 5) visualizes correlations between variables: physical tests located in the same part of the loading plot are correlated. The score plot and the loading plot communicate: subjects located in the same area in the score plot as variables in the loading plot have a high performance within these tests. UB: Upper body, LB: Lower body.

**Fig 2:**
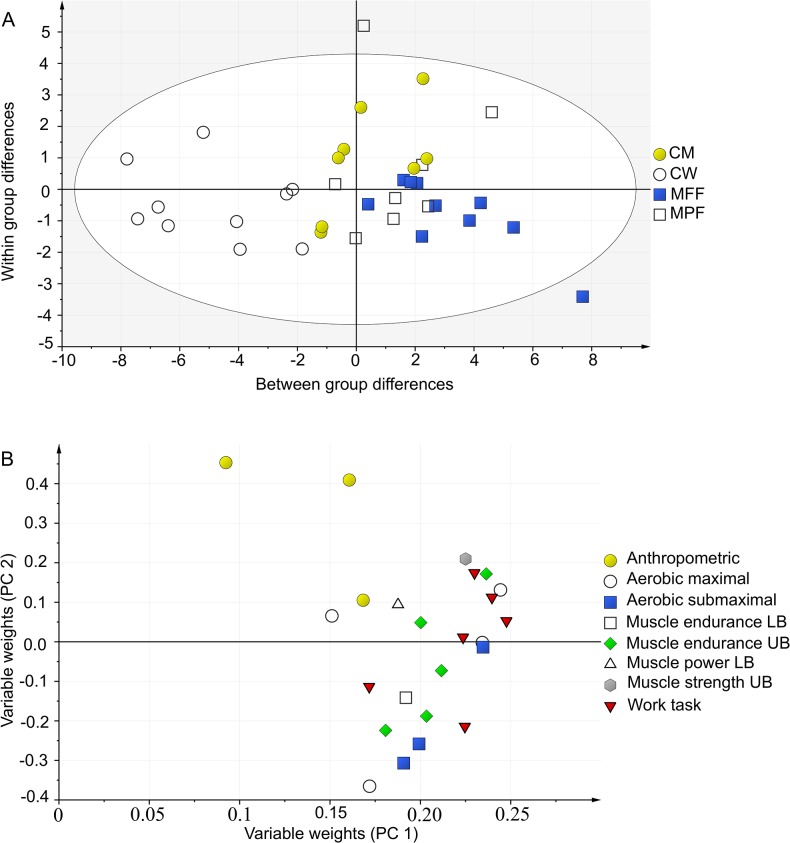
Principal component analysis, field tests and anthropometrics. Score scatter plot (A) of the training-set visualizes the main variation in the data, here seen as between-group differences, and within-group differences. CM: Civilian Men, CW: Civilian Women, MFF: Male Full time Firefighters, MPF: Male Part time Firefighters, n = 36. Loading scatter plot (B: X = 18, Y = 5) visualizes correlations between variables: physical tests located in the same part of the loading plot are correlated. The score plot and the loading plot communicate: subjects located in the same area in the score plot as variables in the loading plot have a high performance within these tests. UB: Upper body, LB: Lower body.

The first and second PCA gives an overview of all included variables (Figs. 1 & 2). The first PCA (Fig. 1 A & B) included laboratory tests, anthropometrics and simulated firefighting work tasks (X = 79, Y = 5) and gave three significant components describing the systematic variation in the data. The second PCA (Fig. 2 A & B) included field tests, anthropometrics and simulated firefighting work tasks (X = 18, Y = 5) and gave two significant components describing the systematic variation in the data.

According to the score scatter plot (Figs. 1 & 2 A) the overall performance for groups of men and women are separated and differences occurs also within subject groups. According to the loading scatter plot (Figs. 1 & 2 B) several work tasks (Table 3) and several physical tests are correlated.

**Table 3:** Correlation matrix, simulated work tasks.

Training-set					
	Stairs	Pulling	Demolition	Rescue	Terrain
**Stairs**	1.0	0.74	0.73	0.83	0.69
**Pulling**		1.0	0.76	0.81	0.66
**Demolition**			1.0	0.73	0.72
**Rescue**				1.0	0.55
**Terrain**					1.0
**Prediction-set**					
	**Stairs**	**Pulling**	**Demolition**	**Rescue**	**Terrain**
**Stairs**	1.0	0.81	0.70	0.81	0.71
**Pulling**		1.0	0.70	0.72	0.67
**Demolition**			1.0	0.72	0.51
**Rescue**				1.0	0.67
**Terrain**					1.0

Bivariate correlations (Pearson r) for simulated work tasks performance time (s): Carrying hose baskets up stairs (Stairs), Hose pulling (Pulling), Demolition at or after a fire (Demolition), Victim rescue (Rescue), Carrying hose baskets over terrain (Terrain). Data is presented for the training-set and for the prediction-set.

The stepwise reductions to the optimal training-set models for Stairs, Pulling, Demolition, and Rescue work capacity are presented in Table 4, and Terrain work capacity is presented in Table 5.

**Table 4:** Variables included for prediction of physical work capacity.

Method	Laboratory model	R^2^/ Q^2^	Field model	R^2^/ Q^2^	EV (R^2^)
**Carrying hose baskets up stairs**					
Step 1:VIP	VO_2max_ (mL∙min^-1^), Maximal Shoulder flexion (W, J), Knee extension (W, J, Nm) and Shoulder extension (W). Endurance Knee extension (W, J) Shoulder extension (W)	0.77/0.75	Rowing 500 m (s), Bench press (n), Maximal hand grip strength (kg), Standing broad jump (m), Barbell shoulder press (n)*, Running 3000 m (s∙kg^−1^)	0.82/0.80	0.81
Step 2: Correlation	VO_2max_ (mL∙min^-1^), Maximal Shoulder flexion and extension (W)	0.78/0.77	Rowing 500 m (s), Standing broad jump (m), Barbell shoulder press (n)*, Running 3000 m (s∙kg^−1^)	0.84/0.82	0.80
**Hose pulling**					
Step 1: VIP	Endurance Shoulder extension (W) and Trunk flexion (J), VO_2max_ (mL∙min^-1^)	0.81/0.77	Bench press (n), Rowing 500 m (s), Maximal hand grip strength (kg), Running 3000 m (s∙kg^−1^), Hand grip endurance (s) *, Standing broad jump (m)	0.79/0.78	0.67
Step 2: Correlation	-	-	Bench press (n), Running 3000 m (s∙kg^−1^), Standing broad jump (m), Handgrip endurance (s) *	0.81/0.80	0.64
**Demolition**					
Step 1: VIP	VO_2max_ (mL∙min^−1^), Maximal Shoulder flexion (J)	0.66/0.59	Bench press (n), Rowing 500 m (s), Running 3000 m (s∙kg^−1^), Maximal handgrip strength (kg)	0.69/0.64	0.38
Step 2: Correlation	-	-	Bench press (n), Running 3000 m (s∙kg^−1^)	0.73/0.68	0.40
**Victim rescue**					
Step 1: VIP	Maximal Shoulder flexion (Nm, W, J) and Knee extension (Nm, J, W), Endurance Knee extension (Nm), Shoulder extension (W) and Trunk flexion (Nm, W), VO_2max_ (mL∙min^−1^), VO_2_ (mL∙min^−1^) at LT	0.75/0.74	Bench press (n), Rowing 500 m (s), Maximal handgrip strength (kg), Standing broad jump (m), Barbell shoulder press (n) *	0.77/0.76	0.69
Step 2: Correlation	Maximal Shoulder flexion (Nm), VO_2max_ (mL∙min^−1^), Endurance Shoulder extension (W)	0.73/0.71	Rowing 500 m (s), Maximal hand grip strength (kg), Barbell shoulder press (n) *	0.75/0.74	0.68

Selection of valid physical tests for prediction of firefighting physical work capacity (n = 36–38) using one laboratory and one field-test model. Before selection, the laboratory model included all laboratory tests and anthropometrics (X = 79); the field model included all field tests and anthropometrics (X = 18). Step 1, Variable Importance for the Projection (VIP): is showing variables included in model after the first selection. Step 2, Correlation: is showing variables included in the model after highly correlated (r ≥ 0.8) variables have been omitted. R^2^ indicates the overall fit of the model, and Q^2^ indicates the predictive power of the model. Field-test models were externally validated (EV) on another subject group (prediction-set: n = 90). VO_2max_: maximal oxygen uptake, J: Joule, NM: Newton meter, % HR_max_: percentage of maximal heart rate used, s∙kg^−1^: time scaled to body mass.

* The test was excluded from the EV because the prediction-set did not do the test.

**Table 5:** Variables included for prediction of Carrying hose baskets over terrain.

Method	Laboratory model	R^2^/Q^2^	Field model	R^2^/ Q^2^	EV (R^2^)
Step 1:VIP	VO_2max_ (mL∙min^−1^ and, mL∙kg-^1^∙min^−1^), OBLA and LT VO_2_ (mL∙min^−1^), Body height (m), VE_max_ (L∙min^−1^), OBLA speed (km∙h^−1^), Maximal and endurance Shoulder flexion (J)	0.72/0.71	Running 3000 m (s), Running 3000 m (s∙kg^−1^), Treadmill walking (% HR_max_) *, Step test (% HR_max_) *, Rowing 500 m (s)	0.78/0.78	0.64
Step 2: Correlation	VO_2max_ (mL∙min^−1^ and mL∙kg-^1^∙min^−1^), Body height (m), OBLA speed (km∙h^−1^)	0.82/0.80	Running 3000 m (s), Running 3000 m (s∙kg^−1^), Treadmill walking (% HR_max_) *, Rowing 500 m (s)	0.82/0.80	0.64

Selection of valid physical tests for prediction of Carrying hose baskets over terrain (n = 38) using one laboratory and one field-test model. Before selection, the laboratory model included all laboratory tests and anthropometrics (X = 79); the field model included all field tests and anthropometrics (X = 18). Step 1, Variable Importance for the Projection (VIP): is showing variables included in model after the first step selection. Step 2, Correlation: is showing variables included in the model after highly correlated (r ≥ 0.8) variables have been omitted. R^2^ indicates the overall fit of the model, and Q^2^ indicates the predictive power of the model. The field-test model was externally validated (EV) on another subject group (prediction-set: n = 90). VO_2max_: maximal oxygen uptake, J: Joule, % HR_max_: percentage of maximal heart rate used, s∙kg^−1^: time scaled to body mass.

* The test was excluded from the EV because the prediction-set did not do the test.

#### Carrying hose baskets up stairs

**Fig 3:**
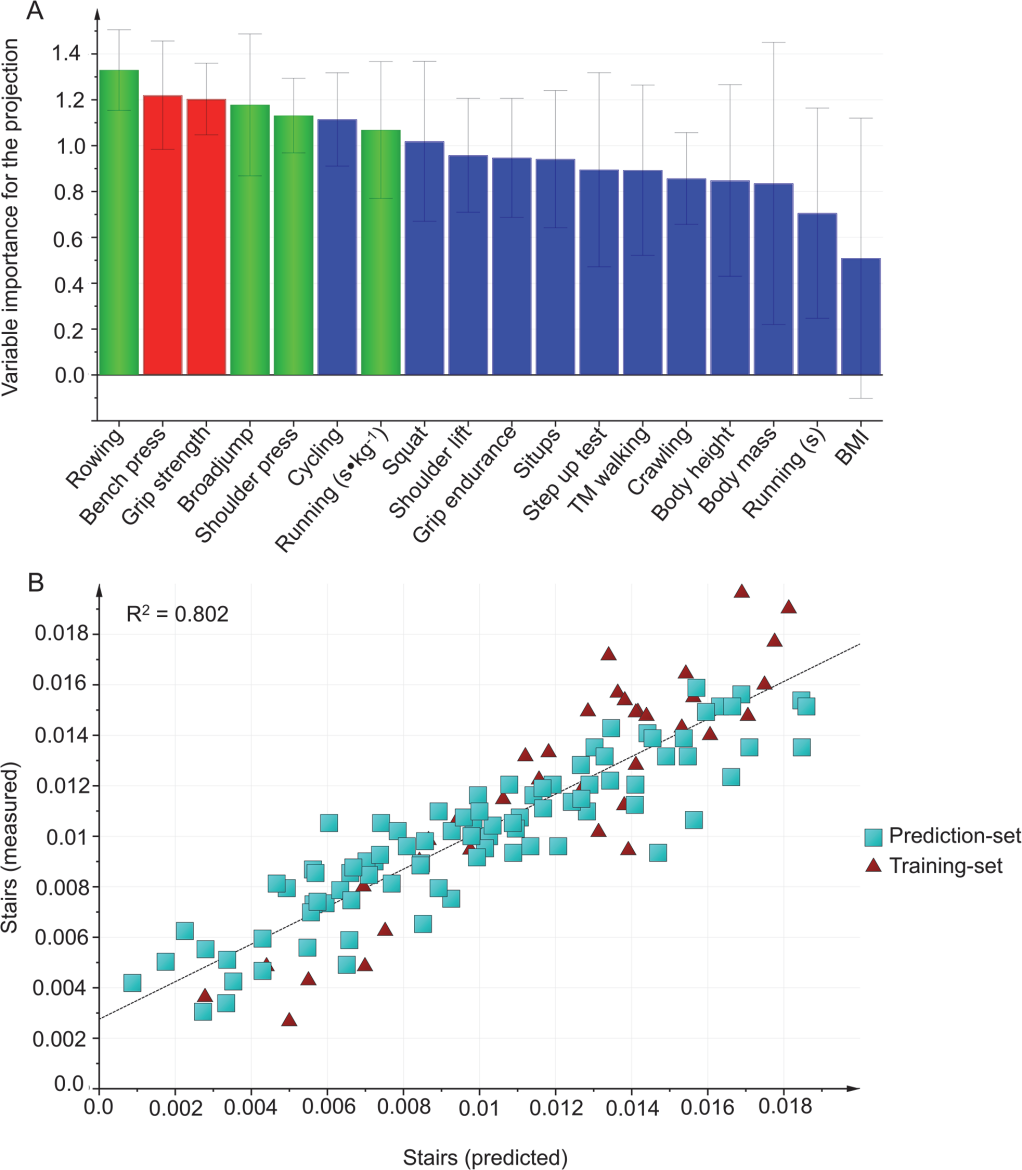
Carrying hose baskets up stairs. A: Variables importance for the projection (VIP) of Carrying hose baskets up stairs work capacity, using the training-set (n = 36). Green bars: Variables included in final the model. Blue bars: Variables were excluded from the model in Step 1, using VIP. Red bars: Variables were excluded from the model in Step 2 because the correlation was r ≥ 0.8 with another included variable. B: External validation is testing the selected model on the prediction-set.

In the first modeling, the best prediction with the highest predictive power included field tests and no anthropometric variables (Table 4, Fig. 3 A & B).

As verified by the external validation (Table 4), the selected field test model was valid with all subjects included (n = 126, R^2^ = 0.80, Q^2^ = 0.79, X = 3), although the *Barbell shoulder press* test was excluded from the model because the prediction-set did not do that test. Also, the model was valid both for firefighters (n = 83, R^2^ = 0.77, Q^2^ = 0.76) and for civilians (n = 43, R^2^ = 0.84, Q^2^ = 0.82).

The selection of physical tests in the second modeling did not change compared to the first modeling. *Rowing 500 m (s)*, *Standing broad jump (m)* and *Running 3000 m (s∙kg* ^-*1*^) remained as the most important variables for prediction of firefighters *Stairs* work capacity.

#### Hose pulling

**Fig 4:**
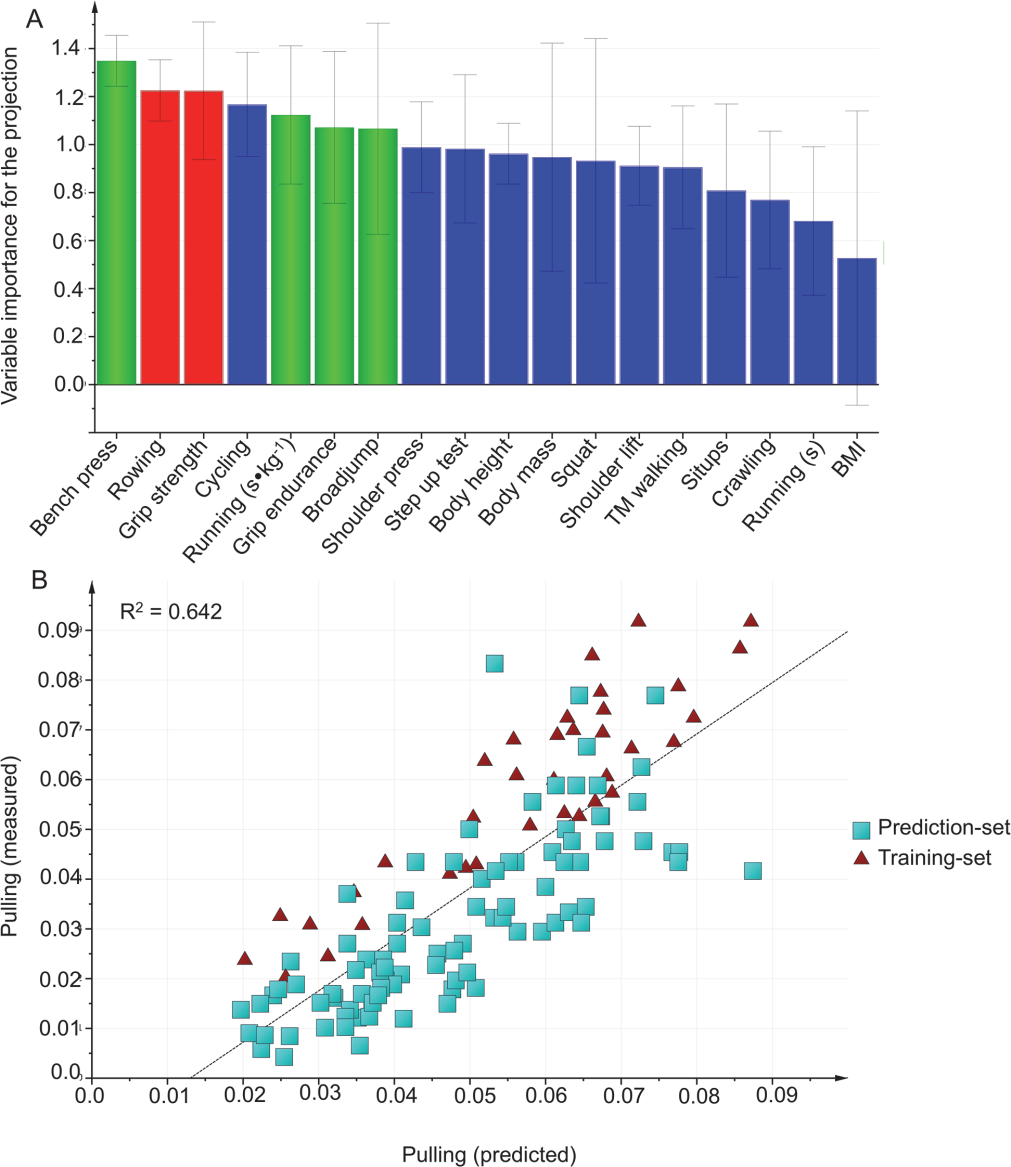
Hose pulling. A: Variables importance for the projection (VIP) of Hose pulling work capacity, using the training-set (n = 37). Green bars: Variables included in the final model. Blue bars: Variables were excluded from the model in Step 1, using VIP. Red bars: Variables were excluded from the model in Step 2 because the correlation was r ≥ 0.8 with another included variable. B: External validation is testing the selected model on the prediction-set.

In the first modeling, the best prediction with the highest predictive power included field tests and no anthropometric variables (Table 4, Fig. 4 A & B).

As verified by the external validation (Table 4), the model was valid with all subjects included (n = 126, R^2^ = 0.65, Q^2^ = 0.64, X = 3), although the prediction-set did not do the *Handgrip endurance* test. Also, the model was valid both for firefighters (n = 83, R^2^ = 0.68, Q^2^ = 0.66) and for civilians (n = 43, R^2^ = 0.84, Q^2^ = 0.82).

In the second modeling, the best prediction with the highest predictive power (n = 126, R^2^ = 0.72, Q^2^ = 0.71) included *Maximal handgrip strength (kg)*, *Bench press (n)*, *Standing broad jump (m)*, *Running 3000 m (s*∙*kg* ^-*1*^), and *body height (m)*. Without body height included in the model, the prediction and predictive power slightly decreased (n = 126, R^2^ = 0.70, Q^2^ = 0.70). Models both including and excluding anthropometrics were valid for firefighters (n = 83, body height included: R^2^ = 0.81, Q^2^ = 0.76, body height excluded: R^2^ = 0.79, Q^2^ = 0.75) and for civilians’ (n = 43, body height included: R^2^ = 0.68, Q^2^ = 0.65, body height excluded: R^2^ = 0.64, Q^2^ = 0.64).

#### Demolition at or after a fire

**Fig 5:**
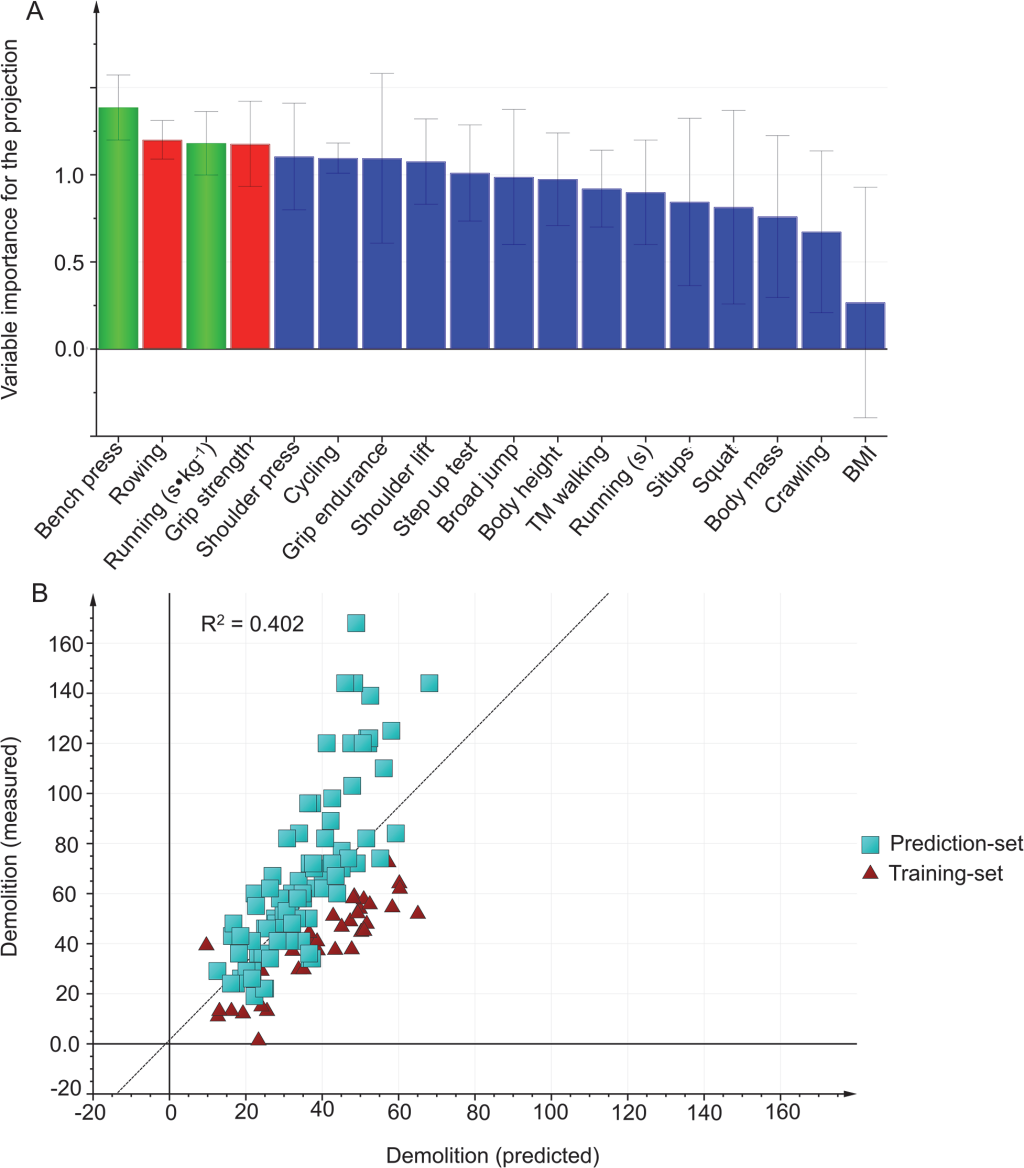
Demolition. A: Variables importance for the projection (VIP) of Demolition work capacity, using the training-set (n = 37). Green bars: Variables included in the final model. Blue bars: Variables were excluded from the model in Step 1, using VIP. Red bars: Variables were excluded from the model in Step 2 because the correlation was r ≥ 0.8 with another included variable. B: External validation is testing the selected model on the prediction-set.

In the first modeling, the best prediction with the highest predictive power included field tests and no anthropometric variables (Table 4, Fig. 5 A & B).

As verified by the external validation (Table 4), the selected field test model was invalid with all subjects included (n = 127, R^2^ = 0.40, Q^2^ = 0.39 X = 2), for firefighters (n = 83), R^2^ = 0.34, Q^2^ = 0.31), and for civilians (n = 43, R^2^ = 0.44, Q^2^ = 0.39).

In the second modeling, the best prediction with the highest predictive power included *Rowing 500 m (s)*, *Maximal handgrip strength (kg)*, *Bench press (n)* and *Running 3000 m (s∙kg* ^-*1*^). The second model was invalid with all subjects included (n = 127, R^2^ = 0.48, Q^2^ = 0.46), for firefighters (n = 83, R^2^ = 0.44, Q^2^ = 0.38), and for civilians (n = 44, R^2^ = 0.45, Q^2^ = 0.40).

#### Victim rescue

**Fig 6:**
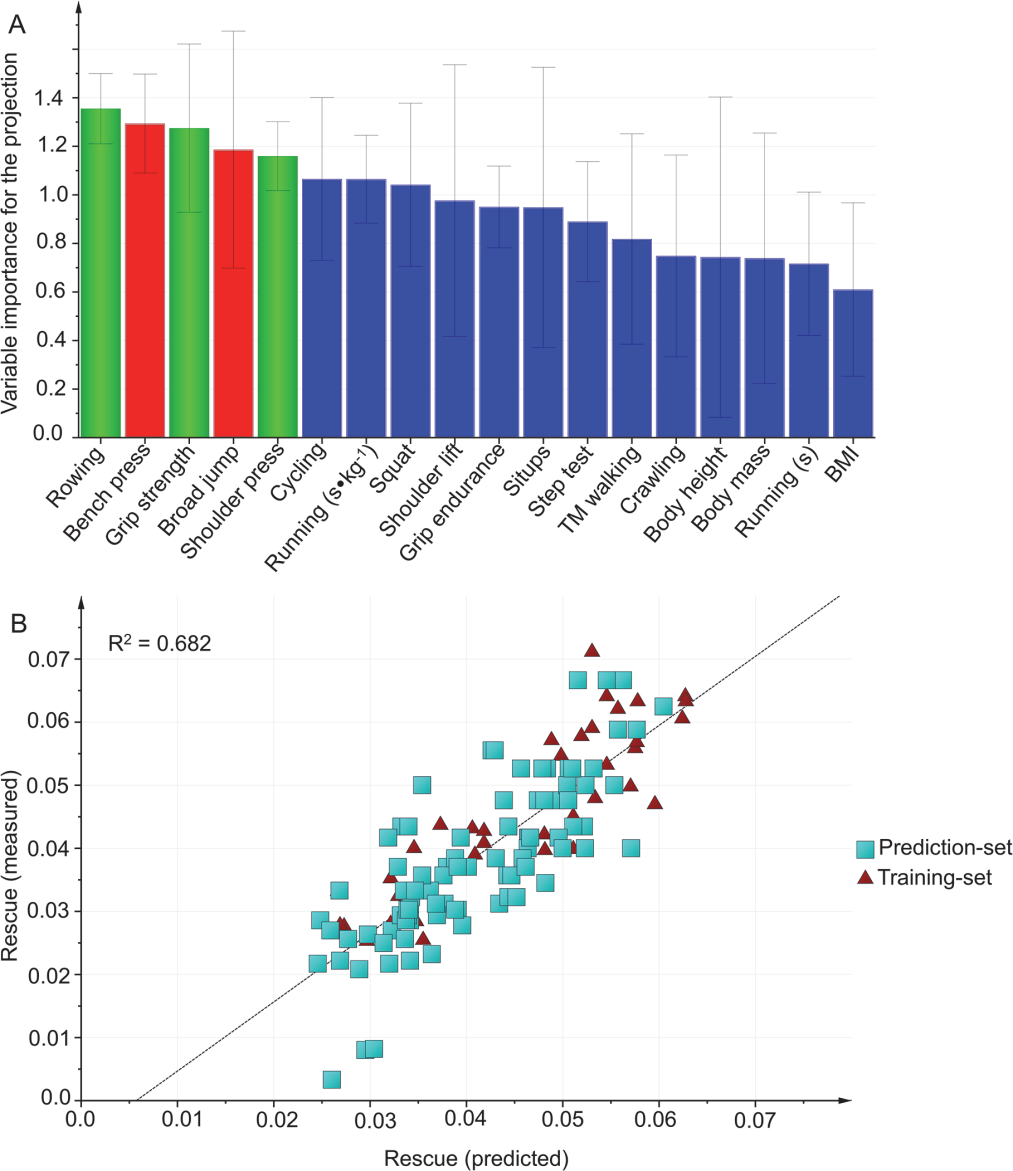
Victim rescue. A: Variables importance for the projection (VIP) of Victim rescue work capacity, using the training-set (n = 37). Green bars: Variables included in the final model. Blue bars: Variables were excluded from the model in Step 1, using VIP. Red bars: Variables were excluded from the model in Step 2, because the correlation was r ≥ 0.8 with another included variable. B: External validation is testing the selected model on the prediction-set.

In the first modeling, the best prediction with the highest predictive power included field tests and no anthropometric variables (Table 4, Fig. 6 A & B).

As verified by the external validation (Table 4), the selected field test model was valid with all subjects included (n = 127, R^2^ = 0.68, Q^2^ = 0.67 X = 2), although the *Barbell shoulder press* test was excluded from the model because the prediction-set did not do that test. Also, the model was valid both for firefighters (n = 83, R^2^ = 0.69, Q^2^ = 0.67) and for civilians (n = 44, R^2^ = 0.64, Q^2^ = 0.61).

In the second modeling, the best prediction with the highest predictive power included *Standing broad jump (m)*, *Rowing 500 m (s)*, *Running 3000 m (s∙kg* ^-*1*^) and *Bench press (n)*. The second model was valid with all subjects included (n = 127, R^2^ = 0.71, Q^2^ = 0.70), for firefighters (n = 83, R^2^ = 0.72, Q^2^ = 0.70) and for civilians (n = 44, R^2^ = 0.68, Q^2^ = 0.66).

#### Carrying hose baskets over terrain

**Fig 7:**
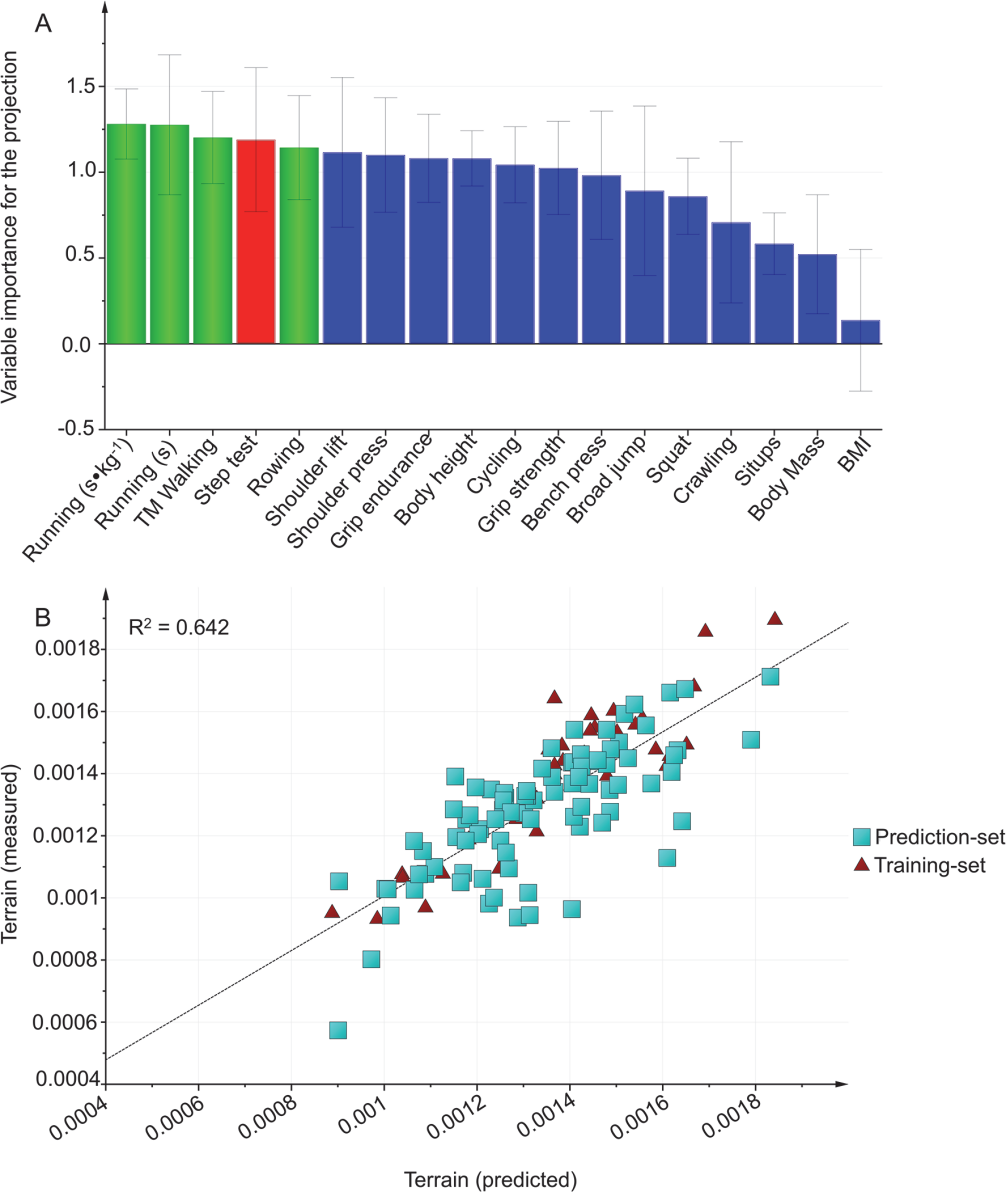
Carrying hose baskets over terrain. A: Variables importance for the projection (VIP) of Carrying hose baskets over terrain work capacity, using the training-set (n = 36). Green bars: Variables included in the final model. Blue bars: Variables were excluded from the model in Step 1, using VIP. Red bars: Variables were excluded from the model in Step 2 because the correlation was r ≥ 0.8 with another included variable. B: External validation is testing the selected model on the prediction-set.

In the first modeling, the prediction and predictive power was equally high with laboratory tests as with field tests (Table 5). The selection of field tests and the external validation for the field-test model is presented in Fig. 7 A & B.

As verified by the external validation (Table 5), the selected field test model was valid with all subjects included (n = 126, R^2^ = 0.65, Q^2^ = 0.63 X = 3), although the prediction-set did not do the *Treadmill walking* test. Also, the model was valid for firefighters (n = 81, R^2^ = 0.66, Q^2^ = 0.63) but not for civilians (n = 45, R^2^ = 0.60, Q^2^ = 0.58).

In the second modeling, the best prediction with the highest predictive power included *Standing broad jump (m)*, *Running 3000 m (s∙kg* ^-*1*^) and *Maximal handgrip strength (kg)*. The second model was valid with all subjects included (n = 127, R^2^ = 0.69, Q^2^ = 0.68), for firefighters (n = 83, R^2^ = 0.71, Q^2^ = 0.70) and for civilians (n = 44, R^2^ = 0.68, Q^2^ = 0.66).

## Discussion

The main finding in this study is that field tests can predict firefighters’ physical work capacity equally well as laboratory tests, and that models excluding anthropometric data are valid for prediction of physical work capacity for firefighting work tasks. Valid models were found with all subjects included, with only firefighters included, and with only civilians included.

### Variables included in the study

#### Work tasks

All studied work tasks have been included in previous studies, but with variations in work rate, time, and external load, when mean or median work time have ranged from 2 min 40 s to 14 min 30 s, active rest periods included [27,28,31,34,35,52–54]. On the extreme end, the Field Walk test and PHT, used for evaluation of wild land firefighters’ work capacity [55] have performance times longer than previously mentioned work task courses: 30 min and 45 min, respectively [56]. The large variation in time and intensity will affect the possibilities to compare studies, due to the differences in contribution of anaerobic and aerobic components on performance.

The cutting a hole in the roof work task (Cutting) was included in previous studies on the same subject group [17,18] but was omitted from the present study because of the low content validity of the test. The performance time was longer compared to a real time situation, the roof was not sloped, and subjects did not wear fire protective clothing.

When physical work capacity is studied, using work tasks that are affected by experience are undesirable. For example, timed ladder climbing [29,30,52,53] may be affected by other capacities than physical, such as technique and acrophobia, and should consequently be either untimed [15] or not included. This study tried to reduce confounding factors, for example by having a chest harness on the dummy being pulled backwards: firefighters’ will have experience of rescuing people while civilians’ may not. Using a chest harness will reduce the influence of technique on performance on this work task, but at the same time decreasing content validity. In the present study, there was no significant difference in work task performance between groups of civilian men and women, and part-time firefighters of the same sex, indicating that physical work capacity, rather than work performance was studied. This finding also indicate that part-time firefighters are not more fit than other civilians, leaving room for future interventions to improve physical work capacity, as well as work performance. Although confounding factors were reduced, other dimensions but physical work capacity may affect performance results, for example mental skills and protective clothing. The present study used equal BA (19 kg) as in the Swedish government treadmill test [37] although lightweight composite BA (12 kg) is used within some municipalities. Others [5,6] have demonstrated that the use of BA and protective clothing reduces physical capacity. Consequently it is impossible to argue that only the dimension physical work capacity was studied.

#### Laboratory tests, field tests and anthropometrics

Firefighters’ measured or predicted aerobic [7,13,27,28,34,38,52,54,56] and anaerobic [28,31,35,56] fitness is well documented, and both laboratory and field tests have previously been studied. Firefighters’ aerobic power is more frequently reported than the aerobic capacity. The reason may be the common use of VO_2max_ predictions, or that aerobic power is assumed to be of higher importance for work capacity than aerobic capacity, as previously suggested by Sharkey and Davis [57].

Firefighters’ maximal muscle strength is most frequently evaluated with maximal hand grip strength [30,31,34–36,38] and measured or predicted one Repetition Maximum (RM) bench press [27,28,30,35]. The mean maximal hand grip strength in studies of firefighters’ work capacity range from 47 to 61 kg for men [30,31,34–36], and 34.5 kg for women [30], showing that the maximal hand grip strength in the present study is similar to previous studies.

Shoulder press [30], bicep curls [30], leg press [27,28,30], leg extension [28], squat [35] and press behind the neck [27], five RM bench press and squat [31] have also been used to study firefighters’ maximal muscle strength. In the present study, none of these physical tests were included; instead maximal muscle strength was measured with Isokinetic laboratory tests. Laboratory tests of firefighters’ muscle strength and endurance are uncommon; we have only found studies of firefighters’ abdominal [35], concentric knee extension [28], and isometric arm lift [33] muscle strength, with knee extension power found not to be important of total performance time in the Candidate Physical Ability Test [28]. Abdominal muscle strength was important for firefighters’ work capacity (Rescue, Stair climb, Rolled hose lift and move, Keiser sled, Hose pull (r = −0.29 to −0.49, p < 0.01 to 0.05) [35] and isometric arm lift was important for the overall assessment of firefighters’ work capacity, in a work task course (R^2^ = 0.49, p < 0.01) [33]. To our knowledge, no one has previously studied a large number of laboratory tests, field tests, and simulated firefighting work tasks on the same subject group of firefighters, using multivariate statistics.

Sit-ups [31,34,35,38], chin-ups [34], bench press [30,31], squat [31], leg press [30], bent over row [31,36], shoulder press [31,36], hand grip [31,36], push up [32,36], wall sit [36], and pull ups [38] have previously been used to determine firefighters’ muscle endurance. Some of these tests were included in the present study. Methodological differences between studies place different demands on muscle work capacity, especially in muscle endurance tests. For example, the barbell weight in the bench press test was lower in the present study (30 kg) compared to a similar study by Rhea et al. [31] (45 kg). Williams- Bell et al. [30] used equal barbell weight as in the present study during the endurance bench press test, but a higher testing speed (30 vs. 25 lifts per minute).

Lindberg et al. [18] previously found that a large proportion of civilian females were not able to do one single chin-up or dips. In Sweden, men and women are aimed to do the same physical tests in recruitment as a firefighter. Although chin-ups ups and dips are common tests in the recruitment of firefighters in Sweden, these tests are unusual in scientific studies of firefighters’ physical work capacity [34]. Due to the risk of discriminating women, chin-ups and dips were therefore not included in the present models. [34]

Anthropometric data, such as body mass, height, BMI and body fat has previously been studied for influence on firefighters’ work capacity [28,30–32,35,38]. Due to the discriminative nature of tests, which cannot be trained, such variables should be excluded when high prediction and predictive power can be achieved with other, not discriminating variables (Table 4 & 5).

Balance is rarely included in studies of firefighters’ physical work capacity, but sometimes in studies of work performance, such as the effect of breathing apparatus [3,58] and the risk to slip and fall [59]. Results from the present study also indicate that balance may not be important (relative to other variables) when predicting work capacity for firefighters.

### Variables selected after modeling and external validation

In an attempt to evaluate firefighters’ physical work capacity by different physical tests estimated, bivariate [17,18,27,28,31,32,34–36,38,52,54,56] and multivariate linear regression [27,28,30,32,34,35,38,52] analyses have previously been used. Bivariate correlations of performance variables including only the training-set data have previously been presented [17,18], although the numbers of variables from laboratory muscle strength and endurance and balance tests are extended in the present study. Bivariate correlation is showing the strength between two variables but does not necessary imply causation. Such statistical analyses are interesting, and also important, for an overview of data. In order to include as few variables as possible in a test battery, bivariate analyses must be supplemented with multivariate statistics. In the present study, models including only field tests were equally good as models including laboratory tests, favoring field tests for being easier to conduct, standardize and perform in local settings.

External validation is one way to test model robustness, and all but one (*Demolition*) first model was valid (Table 4). The low external validation of this work task is difficult to explain because both the prediction and the predictive power were high with all selected field tests included. Also, no significant differences in physical capacity were found between the training-set and the prediction-set for physical tests included in the model (Table 2). In order to test the model robustness of the second model, further studies are needed.

Laboratory tests are complicated and expensive, thus the opportunity of equal physical testing of firefighters at different locations will decrease. Davis et al. [34] found better models for evaluation of firefighters’ general physical work capacity when both laboratory and field tests were included (R^2^ = 0.9) compared to using field tests only (R^2^ = 0.54). Because laboratory tests of firefighters’ work capacity are uncommon, and our models using field tests had equal power to models with laboratory tests, the discussion below primarily focuses on field tests.

A key laboratory test is aerobic fitness, in the present study measured as absolute and relative VO_2max_. In accordance with some [13,27,28,30,36,38,53,54], and opposite to Harvey et al. [52], a high VO_2max_ was found to be important for a high physical work capacity (Table 4 & 5). Harvey et al. [52] found peak arm work rate (W) to be a better predictor (r^2^ = 0.46, p < 0.01) than VO_2max_ (absolute (mL∙kg^-1^: r = 0.1 for men and 0.001 for women, p > 0.05) relative (mL∙kg^-1^∙min^-1^: r^2^ = 0.004 for men and 0.04 for women, p > 0.05) with work capacity on a work task circuit. The conflicting results demonstrate the necessity to execute multivariate modeling in order to weigh aerobic power and capacity in relation to other performance indicators.

The multivariate analysis of field tests in the present study identified Rowing 500 m (s) as an important variable for all work tasks (Table 4 & 5), and Rowing 500 m is also correlated with VO_2max_ in L∙min^-1^ (r_s_ = −0.84, p < 0.01) [17]. The importance of rowing performance for work capacity may depend on the use of both arms and legs, also explaining the results found by Harvey et al [52].

No previously validated test of anaerobic fitness was included in this study, although it is important for firefighters’ work capacity [28,31]. However, anaerobic energy output predominates the aerobic during maximal exercise lasting 0–2 minutes, when large muscle groups are used [60]. Thus, it can be argued that 500 m maximal rowing includes a large anaerobic component, especially in the beginning of the test. Anaerobic power, such as tested by the standing broad jump test was in accordance with others [28] important for firefighters’ work capacity, and when used in combination with other physical tests, an important predictive factor of firefighters’ Rescue, Pulling and Stairs work capacity. Submaximal treadmill walking (% HR_max_) was selected as an important variable for prediction of *Terrain* work capacity. The prediction-set did not perform this test, and the external validation decreased without this test included (Table 5). In a real time situation, such as during recruitment selection testing, knowing the true HR_max_ is impossible, and inclusion of such variable if only estimated (i.e. HR_max_ = 220—age) may give an incorrect assessment of work capacity. It is suggested that a submaximal treadmill walking test [37] can be included as a pass or fail test when testing firefighters’ work capacity.

We and others [30,31,34–36,38] have found maximal hand grip strength, bench press endurance [31], and shoulder muscle endurance [31] to be important for firefighters’ physical work capacity, while Sheaff et al. [28] did not find bench press endurance to be of importance. The reason may be the slightly different method used: In the study by Sheaff et al. [28], subjects were in seated position and an air-powered training machine was used, not in line with the present study. Based on the results in the present study, upper body muscle strength and endurance is important for firefighters’ work capacity, and included tests based on the multivariate modeling are maximal handgrip strength, handgrip endurance, barbell shoulder press, and bench press endurance. The prediction-set did not do all these tests, and the external validation decreased without these tests in the first model.

Results from the present study demonstrate that valid evaluations of work capacity can be achieved primarily from field tests.

Traditionally, physical work capacity is the most studied dimension for firefighters’ work performance. A high physical work capacity is deemed important for a high work performance [7,13–18,27,28,30–32,35], for the safety of the individual firefighter, the colleague, and the victim. If more focus was directed on developing firefighters’ equipment, protective clothing, and division of labor, thus reducing the workload, less focus could be developed on physical capacities.

### Limitations

The low number of women in the training-set, with none of them working as a firefighter, was a known limitation. Because developed models were applicable also on the prediction-set, which included women, our concerns were unfounded and the models are not discriminative for sex.

Both when bivariate and multivariate correlations are used, a high correlation requires a certain distribution of data. We are aware that the high predictions in the present study may be viewed as a result of performance differences between men and women, due to the fact that men as a group perform better than women. One can also assume that subjects of both sexes with an above-average physical capacity are more likely to participate. Consequently, a wide range of performance results is a necessity, and the difference in results between men and women is only an issue if gender-specific tests were to be developed. This was not the case in the present study, and if both physically trained and untrained men had participated, the results would most likely have been the same with respect to correlations and predictions.

The method used during the *Pulling* work tasks was not identical for the training-set and the prediction-set, and may be one reason for the large differences in performance time between subject groups (Table 2). Although both the pulling resistance at full length and the diameter of the rope and hose was the same, using a not fully filled hose makes it harder to grip than a rigid rope, partly due to water oscillation.

All tests of isokinetic muscle strength and endurance consists of isolated, open chain contraction, often single-jointed and thus not functional. In a real time work situation, both concentric and eccentric work is included, and movements often closed-chain, leading to a questionable face and content validity [61].

Because no familiarization of any physical tests and simulated work tasks were allowed, one may argue that lack of familiarization affects the results. On the other hand, tests were developed not to be technically dependent and fit also for non-firefighters. Because only one laboratory test of balance was included in the present study, although field tests are available [3,58,62,63], future studies should include also field tests of this parameter.

## Conclusion

By applying multivariate statistical models, we found valid field tests for prediction of physical work capacity in firefighting work tasks, and can conclude that field test can be used instead of more elaborate laboratory tests. Rowing 500 m (s), maximal hand grip strength, (kg) endurance bench press (n), running 3000 m (s and s∙kg^-1^), barbell shoulder press (n), standing broad jump (m), submaximal treadmill walking (% HR_max_) and handgrip endurance are valid physical tests for prediction of physical work capacity for firefighters.

## References

[1] Sonnentag S. Psychological management of individual performance Chichester: Wiley; 2002.

[2] Koopmans L, Bernaards CM, Hildebrandt VH, Schaufeli WB, de Vet Henrica CW, van der Beek AJ. Conceptual frameworks of individual work performance: a systematic review. J Occup Environ Med. 2011;53: 856–866. 10.1097/JOM.0b013e318226a763 21775896

[3] Son SY, Bakri I, Muraki S, Tochihara Y. Comparison of firefighters and non-firefighters and the test methods used regarding the effects of personal protective equipment on individual mobility. Appl Ergon. 2014;45:1019–1027. 10.1016/j.apergo.2013.12.006 24462474

[4] Dreger RW, Jones RL, Petersen SR. Effects of self-contained breathing apparatus and fire protective clothing on maximal oxygen uptake. Ergonomics 2006;49: 911–920. 16803723 10.1080/00140130600667451

[5] Taylor N, Lewis M, Notley S, Peoples G. A franctionation of the physiological burden of the personal protective equipment worn by fire-fighters. Eur J Appl Physiol. 2012;112: 2913–2921. 10.1007/s00421-011-2267-7 22143844

[6] Hooper A, Crawford J, Thomas D. An evaluation of physiological demands and comfort between the use of conventional and lightweight self-contained breatning apparatus. Appl Ergon. 2001;32: 399–406. 11461041 10.1016/s0003-6870(01)00007-2

[7] O'Connell ER, Thomas PC, Cady LD, Karwasky RJ. Energy costs of simulated stair climbing as a job-related task in fire fighting. J Occup Med. 1986;28: 282–284. 3701477

[8] Kluth K, Pauly O, Keller E, Strasser H. Muscle strain associated with operating three models of fire nozzles and subjective assessment of their ergonomic quality. Occupational Ergonomics 2004;4: 89–104.

[9] Gentzler M, Stader S. Posture stress on firefighters and emergency medical technicians (EMTs) associated with repetitive reaching, bending, lifting and pulling tasks. Work 2010;37: 227–239. 10.3233/WOR-2010-1075 20978330

[10] Bakri I, Lee JY, Nakao K, Wakabayashi H, Tochihara Y. Effects of firefighters' self-contained breathing apparatus' weight and its harness design on the physiological and subjective responses. Ergonomics 2012;55: 782–791. 10.1080/00140139.2012.663506 22506725

[11] Gavhed D, Holmer I. Thermoregulatory responses of firemen to exercise in the heat. Eur J Appl Physiol Occup Physiol. 1989;59: 115–122. 2583139 10.1007/BF02396588

[12] Oksa J, Rintamäki H, Takatalo K, Mäkinen T, Lusa S, Lindholm H, et al Firefighters muscular recovery after a heavy work bout in the heat. Appl Physiol Nutr Metab. 2013;38: 292–299. 10.1139/apnm-2012-0180 23537021

[13] Gledhill N, Jamnik V. Characterization of the physical demands of firefighting. Can J Sport Sci. 1992;17: 207–213. 1325260

[14] Barr D, Gregson W, Reilly T. The thermal ergonomics of firefighting reviewed. Appl Ergon. 2010;41: 161–172. 10.1016/j.apergo.2009.07.001 19664755

[15] Gledhill N, Jamnik VK. Development and validation of a fitness screening protocol for firefighter applicants. Can J Sport Sci. 1992;17: 199–206. 1325259

[16] Henderson ND, Berry MW, Matic T. Field measures of strength and fitness predict firefighter performance on physically demanding tasks. Pers Psychol. 2007;60: 431–473.

[17] Lindberg A-S, Oksa J, Gavhed D, Malm C. Field tests for evaluating the aerobic work capacity of firefighters. PLOS one 2013;8: e68047–e68047. 10.1371/journal.pone.0068047 23844153 PMC3699487

[18] Lindberg A-S, Oksa J, Malm C. Laboratory or field tests for evaluating firefighters' work capacity. Public Library of Science 2014;9: e91215–e91215. 10.1371/journal.pone.0091215 24614596 PMC3948787

[19] Huang CJ, Webb HE, Garten RS, Kamimori GH, Evans RK, Acevedo EO. Stress hormones and immunological responses to a dual challenge in professional firefighters. Int J Psychophysiol. 2010;75: 312–318. 10.1016/j.ijpsycho.2009.12.013 20079388

[20] Henderson ND, Berry MW, Matic T. Predicting long-term firefighter performance from cognitive and physical ability measures. Pers Psychol. 2010;63: 999–1039.

[21] Lusa S, Punakallio A, Luukkonen R, Louhevaara V. Factors associated with changes in perceived strain at work among fire-fighters: a 3-year follow-up study. International Archives of Occupational & Environmental Health 2006;79: 419–426. 10.1016/S2213-8587(14)70178-0 16331518

[22] Lindberg AS, Malm C, Oksa J, Gavhed D. Self-rated physical loads of work tasks among firefighters. Int J Occup Saf Ergon. 2014;20: 309–321. 24934427 10.1080/10803548.2014.11077042

[23] Lusa S, Louhevaara V, Kinnunen K. Are the job demands on physical work capacity equal for young and aging firefighters. J Strength Cond Res. 1994;36: 70–74. 8138852

[24] Garver JN, Jankovitz KZ, Danks JM, Fittz AA, Smith HS, Davis SC. Physical fitness of an industrial fire department vs. a municipal fire department. J Strength Cond Res. 2005;19: 310–317. 15903368 10.1519/R-14934.1

[25] Phillips M, Payne W, Lord C, Netto K, Nichols D, Aisbett B. Identification of physically demanding tasks performed during bushfire suppression by Australian rural firefighters. Appl Ergon. 2012;43: 435–441. 10.1016/j.apergo.2011.06.018 21802652

[26] Perroni F, Cignitti L, Cortis C, Capranica L. Physical fitness profile of professional Italian firefighters: differences among age groups. Appl Ergon. 2014;45: 456–461. 10.1016/j.apergo.2013.06.005 23849328

[27] von Heimburg ED, Rasmussen AK, Medbø JI. Physiological responses of firefighters and performance predictors during a simulated rescue of hospital patients. Ergonomics 2006;49: 111–126. 16484140 10.1080/00140130500435793

[28] Sheaff AK, Bennett A, Hanson ED, Kim Y-S, Hsu J, Shim JK. Physiological determinants of the candidate physical ability test in firefighters. J Strength Cond Res. 2010;24: 3112–3122. 10.1519/JSC.0b013e3181f0a8d5 20938354

[29] Dreger RW, Petersen SR. Oxygen cost of the CF–DND fire fit test in males and females. Appl Physiol Nutr Metab. 2007;32: 454–462. 17510680 10.1139/H07-020

[30] Williams-Bell FM, Villar R, Sharratt MT, Hughson RL. Physiological demands of the firefighter Candidate Physical Ability Test. Med Sci Sports Exerc. 2009;41: 653–662. 10.1249/MSS.0b013e31818ad117 19204584

[31] Rhea MR, Alvar BA, Gray R. Physical fitness and job performance of firefighters. J Strength Cond Res. 2004;18: 348–352. 15142006 10.1519/R-12812.1

[32] Michaelides MA, Parpa KM, Thompson J, Brown B. Predicting Performance on a Firefighter's Ability Test From Fitness Parameters. Res Q Exerc Sport 2008;79: 468–475. 19177948 10.1080/02701367.2008.10599513

[33] Sothmann MS, Gebhardt DL, Baker TA, Kastello GM, Sheppard VA. Performance requirements of physically strenuous occupations: validating minimum standards for muscular strength and endurance. Ergonomics 2004;47: 864–875. 15204279 10.1080/00140130410001670372

[34] Davis PO, Dotson CO, Santa Maria DL. Relationship between simulated fire fighting tasks and physical performance measures. Med Sci Sports Exerc. 1982;14: 65–71. 7070261 10.1249/00005768-198201000-00013

[35] Michaelides MA, Parpa KM, Henry LJ, Thompson GB, Brown BS.Assessment of physical fitness aspects and their relationship to firefighters' job abilities. J Strength Cond Res. 2011;25: 956–965. 10.1519/JSC.0b013e3181cc23ea 20703167

[36] Phillips M, Petersen A, Abbiss CR, Netto K, Payne W, Nichols D. Pack Hike Test finishing time for Australian firefighters: Pass rates and correlates of performance. Appl Ergon. 2011;42: 411–418. 10.1016/j.apergo.2010.08.020 20888552

[37] Arbetsmiljöverket:Medicinska kontroller i arbetslivet. Arbetsmiljöverkets föreskrifter om medicinska kontroller i arbetslivet och allmänna råd om tillämpningen av föreskrifterna Solna: Swedish Work Environment Authority 2005: 18–19.

[38] Williford HN, Duey WJ, Olson MS, Howard R, Wang N. Relationship between fire fighting suppression tasks and physical fitness. Ergonomics 1999;42: 1179–1186. 10503052 10.1080/001401399185063

[39] Eriksson L, Byrne T, Johansson E, Trygg J, Vikström C. Multi- and Megavariate Data Analysis Basic principles and Applications. Third revised edition: Umetrics Academy; 2013.

[40] Malm C, Lindberg A-S, Stene F. Brandmannens fysiska förmåga. Delrapport 2, Fysiologiska tester. Karlstad: Räddningsverket; 2005.

[41] Levels K, de Koning JJ, Mol E, Foster C, Daanen HA. The effect of pre-warming on performance during simulated firefighting exercise. Appl Ergon. 2014;45: 1504–1509. 10.1016/j.apergo.2014.04.011 24816137

[42] DeBlasi F. Isokinetic testing and data interpretation—data analysis Biodex Medical System, Inc. 2003.

[43] Brown LE, Weir JP. ASEP procedures recommendation 1: Accurate assessment of muscular strength and power. JEPonline 2001;4: 21.

[44] Bergkvist M, Hedberg G, Rahm M. Utvärdering av test för bedömning av styrka, rörlighet och koordination Solna: Arbetsmiljöinstitutet; 1992.

[45] Mc Ardle W, F K, Katch V. Exercise physiology: energy, nutrition and human performance Pennsylvania, USA: Lippincott Williams & Wilkins; 2001.

[46] Tokmakidis S, Leger L. Could the fixed blood lactate points represent the threshold and correlate well with performance? Coaching & Sport Science Journal 1995;1: 19–24.

[47] Sjodin B, Jacobs I. Onset of blood lactate accumulation and enzyme activities in m. vastus lateralis in man. Int J Sports Med. 1981;2: 166–170. 6460707 10.1055/s-2008-1034605

[48] Lund A, Lund M.2013. Testing for normality: Laerd statistics [cited 20 May 2013]. Available: https://statistics.laerd.com/premium/tfn/testing-for-normality-in-spss.php

[49] Trygg J, Wold S. Orthogonal projections to latent structures (O-PLS). Journal of Chemometrics 2002;16: 119–128.

[50] Wold S, Esbensen K, Geladi P. PRINCIPAL COMPONENT ANALYSIS. Chemometrics and Intelligent Laboratory Systems 1987;2: 37–52.

[51] Stone M. CROSS-VALIDATORY CHOICE AND ASSESSMENT OF STATISTICAL PREDICTIONS. Journal of the Royal Statistical Society Series B-Statistical Methodology 1974;36: 111–147.

[52] Harvey DG, Kraemer JL, Sharratt MT, Hughson RL. Respiratory gas exchange and physiological demands during a fire fighter evaluation circuit in men and women. Eur J Appl Physiol. 2008;103: 89–98. 10.1007/s00421-008-0673-2 18204853

[53] Sothmann M, Saupe K, Jasenof D, Blaney J, Donahue Fuhrman S, Woulfe T. Advancing age and the cardiorespiratory stress of fire suppression: Determining minimum standards for aerobic fitness. Human performance 1990;3: 217–236.

[54] Louhevaara V, Soukainen J, Lusa S, Tulppo M, Tuomi P, Kajaste P. Development and evaluation of a test drill for assessing physical work capacity of fire-fighters. Int J Ind Ergon. 1994;13: 139–146.

[55] United States Department of Agriculture, US Forest Service.2002. The Pack Test” Work Capacity Testing for Wildland Firefighters: Ensuring Wildland Firefighter Safety [cited 15 October 2014]. Available:. http://www.fs.fed.us/fire/safety/wct/2002/pack_test_info_sheet.pdf

[56] Lord C, Netto K, Petersen A, Nichols D, Drain J, Phillips M. Validating ‘fit for duty’ tests for Australian volunteer fire fighters suppressing bushfires. Appl Ergon. 2012;43: 191–197. 10.1016/j.apergo.2011.05.003 21714952

[57] Sharkey BJ, Davis PO. Hard work: defining physical work performance requirements Champaign, IL: Human Kinetics; 2008

[58] Punaxallio A, Lusa S, Luukkonen R. Protective equipment affects balance abilities differently in younger and older firefighters. Aviat Space Environ Med. 2003;74: 1151–1156. 14620471

[59] Punakallio A, Hirvonen M, Grönqvist R. Slip and fall risk among firefighters in relation to balance, muscular capacities and age. Saf Sci. 2005;43: 455–468.

[60] Åstrand P-O, Rodahl K, Dahl H, Stromme S. Textbook of Work Physiology, Fourth edition USA: Human Kinetics; 2003.

[61] Face Validity. alleydog.com. 2013. [cited 24 October 2014]. Available: http://www.alleydog.com/glossary/definition.php?term=Face%20Validity

[62] Schneiders AG, Sullivan SJ, Gray AR, Hammond-Tooke GD, McCrory PR. Normative values for three clinical measures of motor performance used in the neurological assessment of sports concussion. J Sci Med Sport 2010;13: 196–201. 10.1016/j.jsams.2009.05.004 19560971

[63] Kong PW, Suyama J, Cham R, Hostler D. The relationship between physical activity and thermal protective clothing on functional balance in firefighters. Res Q Exerc Sport 2012;83: 546–552. 23367817 10.1080/02701367.2012.10599144 PMC4895198

